# Late endovascular coil migration following traumatic pulmonary artery pseudoaneurysm embolization: case report

**DOI:** 10.1186/s13019-022-01841-7

**Published:** 2022-04-27

**Authors:** Alina-Maria Budacan, Akshay J. Patel, Helen Foss, Valeria Abiuso, Arul Ganeshan, Maninder Kalkat

**Affiliations:** 1 Department of Thoracic Surgery, Queen Elizabeth Hospital, University Hospitals Birmingham, Mindelsohn Way, Birmingham, B15 2TH UK; 2 Institute of Immunology and Immunotherapy, University of Birmingham, Birmingham, UK; 3 Department of Interventional Radiology, University Hospitals Birmingham (Heart of England), Bordesley Green East, Birmingham, B9 5SS UK

**Keywords:** Endobronchial coil, Embolization, Complications

## Abstract

**Background:**

Percutaneous vascular interventions are performed for the treatment of haemoptysis and involve embolization of bronchial arteries, pulmonary arteries and pulmonary arteriovenous malformations. There are isolated reports of embolization of pseudoaneurysms forming in the pulmonary vasculature. The migration of components of the coils used in the embolization of vascular pulmonary pathologies is rare.

**Case presentation:**

A 46-year-old man presented to the emergency department with cough, haemoptysis, and expectoration of lengths of metal wire. He had an episode of coughing out a wire about a year prior to his admission to our hospital, which he attributed to be present in the can of coke he had consumed at that time and did not report it to the doctors. His past medical history was significant for stab injury to the right chest 17 years ago, for which he underwent right thoracotomy and exploration for bleeding. Injury to the lung parenchyma was noted and repair was performed by suturing the defect. Post operatively the CT scan demonstrated development of pulmonary artery pseudoaneurysm. We report a case of a patient expectorating coils 17 years after embolization of this traumatic pulmonary artery pseudoaneurysm. Radiological imaging demonstrated coils in the perihilar area of the lung parenchyma and in the tracheobronchial lumen. Operative intervention was used to remove the coils.

**Conclusions:**

Although percutaneous catheter based vascular interventions have emerged as safe and effective procedures, the long-term complications such as coil migration, recanalization and need for further embolization ought to be considered and patients need to be counselled and followed-up accordingly. To the best of our knowledge, this is the first case of migrated coil post embolization of post-traumatic pulmonary artery pseudoaneurysm. Ultimately, the management of endobronchial coil migration post embolization, be it surgical or bronchoscopic, should be decided on a case-by-case basis, considering the patient’s symptoms and the risk fatal complications.

## Introduction

Post-traumatic pulmonary artery pseudoaneurysms are rare, at risk of enlargement and rupture, and associated with high mortality [1]. They can be managed surgically or via percutaneous endovascular interventions, such as introduction of metallic coils, with reported good outcomes [2]. Despite having a low rate of complications, coil embolization for pulmonary artery disorders do pose some risks such as recanalization, coil migration, coils misplaced in undesirable sites, damage to the vessel wall and delayed bacterial contamination of the thrombus and pulmonary artery. Erratic coil migration into the bronchial tree or other areas of the lung parenchyma is a very rare complication, with only a few cases having been described in the literature [3–5]. Erosion into the bronchi results in cough, expectoration, haemoptysis, and hoarseness [3]. The fistulisation into the pulmonary artery can result in air embolism or life-threatening haemoptysis [6, 7].

## Case report

We report the case of a patient who expectorated a coil 17 yrs following embolization of a post-traumatic pulmonary artery pseudoaneurysm. A 46-year-old man presented to the emergency department with a cough, haemoptysis, and expectoration of lengths of metal wire. 17 years previously he had suffered a significant stab injury to the chest, for which he underwent right thoracotomy and exploration in a different hospital. A suture repair of a parenchymal lung injury was performed. Post-operative computed tomography (CT) scan demonstrated pulmonary artery pseudoaneurysm formation and percutaneous endovascular coils were inserted to prevent rupture and massive haemoptysis. The medical records had been destroyed by the time of this presentation; hence no further information was available.

**Figure. 1:**
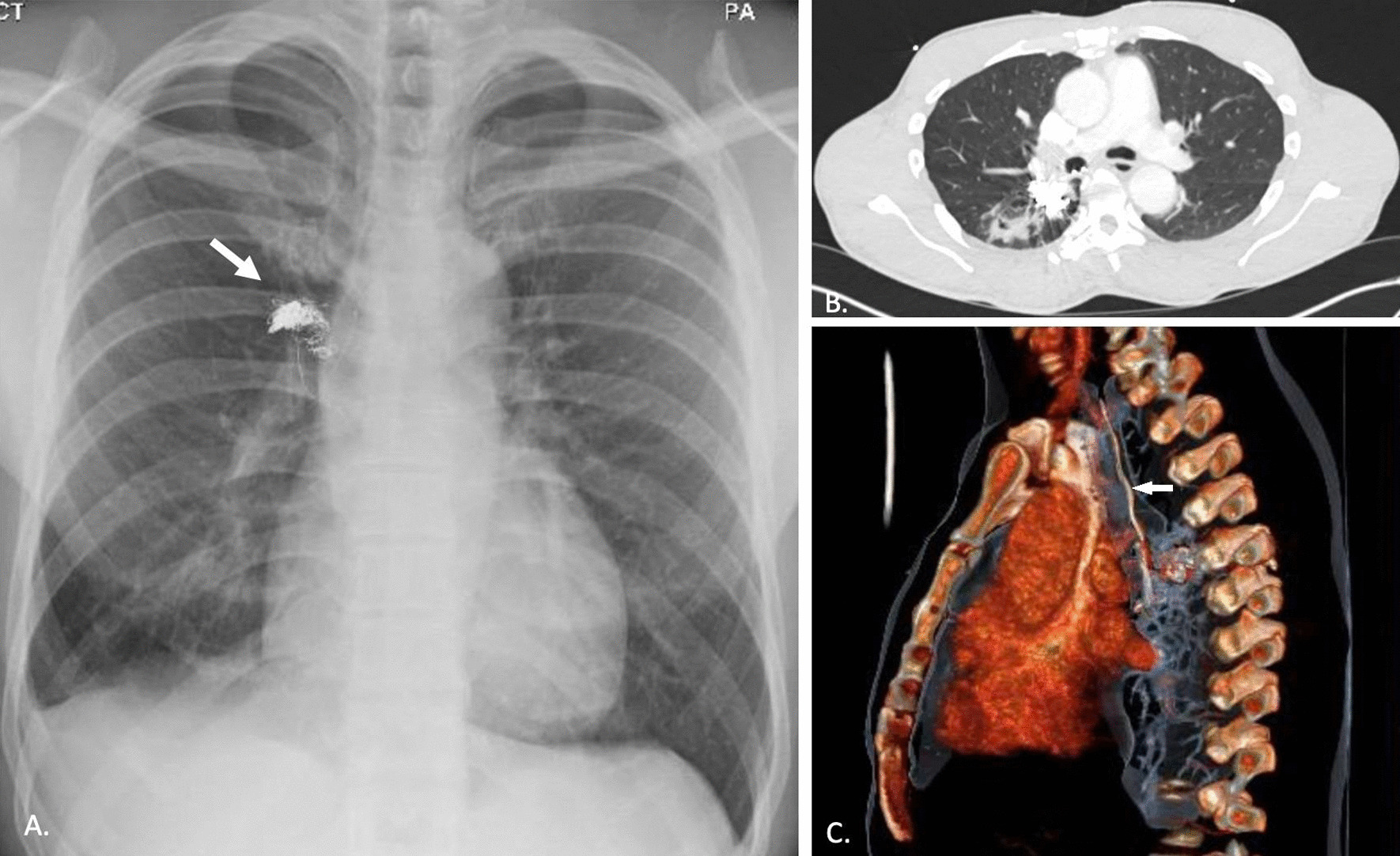
Radiological findings: **A** Chest x-ray showing the coils in the right chest (arrow). **B** Axial view confirming bunch of coils lying posterior to the hilum. Associated changes in the lung parenchyma are visible. **C** Reconstructed CT scan images demonstrating a coil extending into right bronchus and up to the larynx (arrow)

During the current admission, chest x-ray and chest CT demonstrated coils in the right lung parenchyma posterior to the oblique fissure, with a length of coil in the lower lobe bronchus, resulting in adjacent haemorrhagic areas and pneumatoceles (Fig. 1). The patient underwent rigid and flexible bronchoscopy, re-do right thoracotomy, lower lobectomy and removal of the coils.

Bronchoscopy identified a thin wire emerging from the right lower lobe basal segmental bronchus into the right main bronchus, extending to the proximal trachea and larynx. There was marked inflammation with significant blood-stained purulent secretions emanating from the lower lobe bronchus. The wire could not be pulled out with gentle traction. On entry into the chest, the lung was densely adherent to the chest wall and posterior mediastinum. The adhesions were particularly dense and inseparable from the oesophagus, azygos vein and the whole area was one thick indistinguishable fibrotic mass. A subsequent lower lobe bronchotomy was performed and the wire was traced into the lower lobe. This wire was removed, and lower lobectomy was performed by dissecting out all the relevant structures. The bronchus intermedius was stapled off just distal to uptake of middle lobe bronchus where it was less inflamed. After removal of the lower lobe, it was noted that there was a dense fibrotic mass around the posterior segment of the upper lobe. The upper lobe itself was densely fibrotic and stuck to the chest posteriorly and inseparable from the oesophagus and the azygos vein.

After lower lobe removal, the residual fibrotic area was incised to ensure no further wires were left in situ. It was well encapsulated (3*2 cm) and this process revealed necrotic pinkish grey material and a bunch of wires which were subsequently removed. At this point the cavity was closed as there was no evidence for fistulation into the airways or any such tracheobronchial communication. Several coils of metal wires were removed, and the cavity was cleared of all the necrotic debris. The wall of this cavity was inflamed granulation tissue and due to the size, location and adhesions to the mediastinum and oesophagus, the resection was felt not to be possible. In view of this, the cavity was closed using interrupted 2-0 Vicryl suture in two layers ensuring obliteration of the cavity.

Four days post-operatively, the patient showed no sign of clinical improvement and this correlated with worsening inflammatory markers and an exaggerated SIRS response. Features of empyema were noted on imaging. The patient was taken back to theatre for rigid bronchoscopy where pus was seen coming out of the right lower lobe bronchial stump soiling the left side. This was vigorously suctioned, and the chest re-opened via the same posterolateral incision (right). The cavity was washed out and it was noted that the previous suture on the capsule of the posterior segment of upper lobe had broken down and resulted in a communication between the capsule and the stump of the lower lobe bronchus. As such, an intercostal muscle bundle was harvested and patched on the opening of the fistulous tract of the lower lobe bronchial stump with multiple 3-0 vicryl. Post patching, there was minimal air leak on dual lung ventilation and at repeat bronchoscopy there was no further purulent discharge from lower lobe stump.

Streptococcus anginosus, Bacteroides fragilis and Eikenella corrodens were grown from operative samples. The patient was discharged home with chest drain in situ and a long course of appropriate antibiotics. The chest drain was later removed in the outpatient clinic. The current follow-up period to date is 12 months since discharge.

## Comment

The patient described in this report proceeded to surgery to mitigate against the potential for complications such as haemoptysis, recurrent chest infections and troublesome symptoms of cough. The type of intervention, however, was debateable between bronchoscopic retrieval of the coils or open surgical procedure to resect the lung parenchyma containing the coils. The uncertainty of retrieving all the coils endoscopically, possibility of haemorrhage and perceived difficulty of emergency thoracotomy due to adhesions from the previous surgery led to the decision in favour of a surgical intervention.

Some cases of migrated coils fistulating into the bronchus have been removed endobronchially using rigid bronchoscopy [3]. However, most documented cases with migration of these coils have been treated with surgical resection of varying amounts of lung parenchyma and coil retrieval. Abad et al. reported a case of endobronchial coil migration 6 weeks after embolization for a segmental pulmonary artery aneurysm, treated by right lower lobectomy [4] and the use of basal segmentectomy has also been reported to treat similar complications [8].

To the best of our knowledge, this is the first case of migrated coil following embolization of post-traumatic pulmonary artery pseudoaneurysm. On reflection, placing a muscle bundle or some form of vascularised pedicle over the bronchial stump at the time of the lobectomy, particularly in the setting of such inflammation may have helped to avoid fistulation and propagation of sepsis. Ultimately, the management of endobronchial coil migration post-embolization, be it surgical or bronchoscopic, should be decided on a case-by-case basis, taking into account the patient’s symptoms and the risk of fatal complications.

## Data Availability

All data and materials are available upon reasonable request from the corresponding author.
